# Ranking’s Half-Yearly Abstract

**Published:** 1847-09

**Authors:** 


### RANKING'S HALF-YEARLY ABSTRACT.

The fifth number of this very valuable publication has come to us enriched with its usual variety of scientific and practical matter. Our readers are aware that the "Abstract" is published by Messrs. Lindsay & Blakiston, in Philadelphia, at the low price of $1 50 per annum. The editor, Dr. Ranking, is assisted by Drs. Guy, Day, Ancell, and Kirkes, whose reports on the progress of the medical sciences contained in this number are worth many times the annual cost of the Abstract. These reports relate to practical medicine, pathology and therapeutics, surgery, chemistry, midwifery, diseases of women and children, anatomy and physiology, forensic medicine, materia medica and pharmacy. We cordially recommend this admirable publication to our readers.

